# Genetic Diversification by Somatic Gene Conversion

**DOI:** 10.3390/genes2010048

**Published:** 2011-01-10

**Authors:** Kohei Kurosawa, Kunihiro Ohta

**Affiliations:** Department of Life Sciences, Graduate School of Arts and Sciences, The University of Tokyo, Komaba 3-8-1, Meguro-ku, Tokyo 153-8902, Japan; E-Mail: cc107708@mail.ecc.u-tokyo.ac.jp

**Keywords:** immunoglobulin locus, gene conversion, DNA break, recombination

## Abstract

Gene conversion is a type of homologous recombination that leads to transfer of genetic information among homologous DNA sequences. It can be categorized into two classes: homogenizing and diversifying gene conversions. The former class results in neutralization and homogenization of any sequence variation among repetitive DNA sequences, and thus is important for concerted evolution. On the other hand, the latter functions to increase genetic diversity at the recombination-recipient loci. Thus, these two types of gene conversion play opposite roles in genome dynamics. Diversifying gene conversion is observed in the immunoglobulin (Ig) loci of chicken, rabbit, and other animals, and directs the diversification of Ig variable segments and acquisition of functional Ig repertoires. This type of gene conversion is initiated by the biased occurrence of recombination initiation events (e.g., DNA single- or double-strand breaks) on the recipient DNA site followed by unidirectional homologous recombination from multiple template sequences. Transcription and DNA accessibility is also important in the regulation of biased recombination initiation. In this review, we will discuss the biological significance and possible mechanisms of diversifying gene conversion in somatic cells of eukaryotes.

## 1. What Is “Gene Conversion”?

Genetic rearrangements play pivotal roles not only in promoting genetic diversity, but also in maintaining genetic integrity. When lesions are introduced into the genomic DNA of somatic cells, DNA breaks must be repaired promptly to prevent chromosomal aberrations or cell death. DNA homologous recombination is an essential process involved in DNA repair, particularly of DNA-double strand breaks (DSBs).

**Figure 1:**
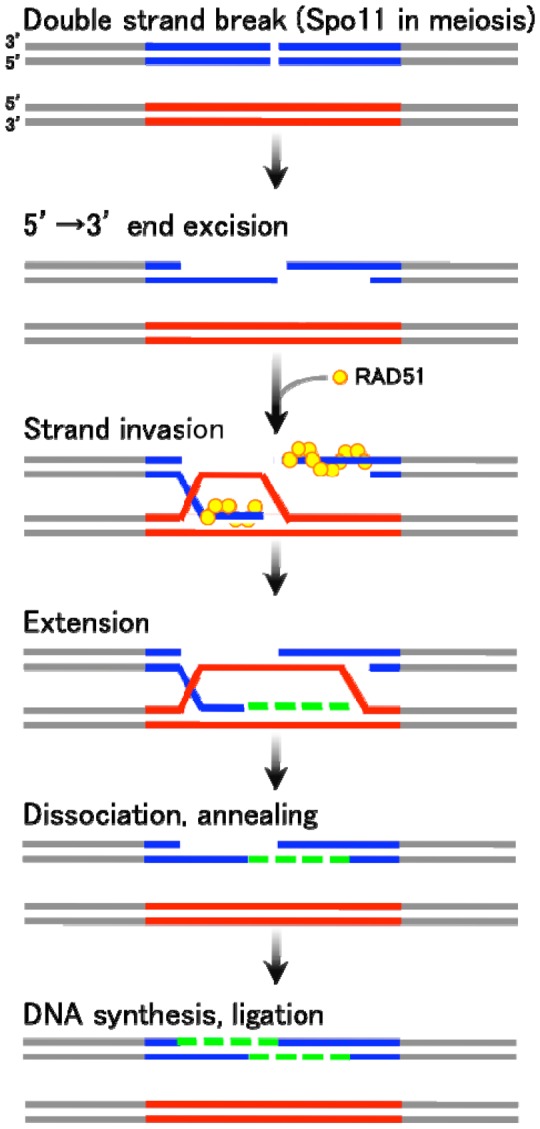
Gene conversion by the Synthesis-Dependent Strand Annealing (SDSA) mechanism.

There are two major types of homologous recombination: gene conversion (non-crossover) and crossover recombination. The former type results in “copy and paste” type non-reciprocal transfer of genetic information, whereas the latter leads to reciprocal exchange between two homologous chromosomes. Gene conversion is mainly triggered by DSB formation, followed by the generation of single-stranded DNA tails with free 3′ ends. These 3′-end DNA tails then invade an intact homologous DNA duplex (Figure 1). The invading single-strand end forms a heteroduplex with a sequence of homologous DNA within the duplex, and further primes DNA replication using unbroken DNA as a template. The newly synthesized DNA end eventually rehybridizes with the original broken DNA molecule by Watson-Crick base pair interaction. This process is called “synthesis-dependent strand annealing (SDSA)” mechanism [1–3] (Figure 1). On the other hand, crossover products, often found in meiotic homologous recombination, are generated via relatively stable and differentiated intermediates displaying double Holliday junctions (DSB repair mechanism) [4].

In somatic cells, gene conversion is assumed to occupy a position in the major homologous recombination pathway, since the induction of a targeted DSB in Chinese hamster ovary cells by the I-SceI endonuclease mainly enhances localized gene conversion events (97%) but not reciprocal recombination [5]. Gene conversion often accompanies unidirectional “copy and paste” type transfer of genetic information between two homologous DNA sequences, which can either be on different chromosomes or on the same chromatid. Because of this, interlocus gene conversion is thought to have the potential to cause genetic diseases [6]. In addition, interallelic gene conversion may sometimes accompany “loss of heterozygosity”, which is one of the major causes of cancer development. Furthermore, gene conversion is pivotal in the “concerted evolution” of duplicated genes, a case in which the sequence variation between duplicated DNA sequences remains low [7–14].

As seen in Figure 1, gene conversion is basically initiated by the DSB formation at the recombination-recipient sites. The DSB ends are processed to have single stranded DNA tails, one of which eventually invades into the duplex of unbroken DNA. The invaded single strand DNA tail then forms a heteroduplex with the homologous DNA stretch in the unbroken template strand. The free DNA end of this heteroduplex primes a repair DNA synthesis. After a strand extension, the newly synthesized strand dissociates form the unbroken template DNA and anneals with the original broken DNA. Finally, the single strand DNA gap is filled followed by a ligation of DNA nicks. In this process, the DNA sequence on the unbroken DNA strand is converted to the broken strand, thereby accompanying a unidirectional transfer of genetic information.

## 2. Diversifying Gene Conversion in Chicken B Cells

**Figure 2:**
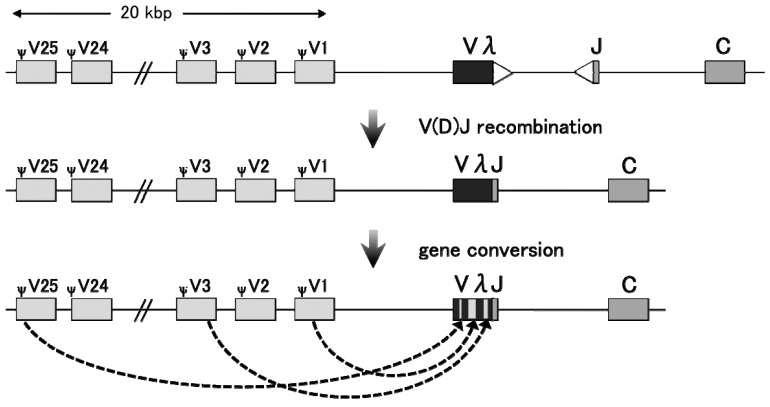
Organization of the chicken immunoglobulin light chain (IgL) locus. The germ line chicken IgL locus consists of a tandem array of pseudo V segments (ψV1–25), Vλ, an intervening sequence with joining signals (open triangles) and a transcription silencer, J and C elements. After V(D)J recombination, a unique functional VλJ segment is generated. The V region in this segment functions as a recipient of successive templated gene conversions from the upstream pseudo V segments, thereby the DNA sequence in the V region is diversified.

Gene conversion tends to negate sequence variation among duplicated genes or DNA sequences; hence, it is important for the concerted evolution of duplicated genes. However, a class of gene conversion functions to increase genetic diversity at regions of frequent recombination. Such gene conversion is observed in the immunoglobulin (Ig) loci of chicken, rabbit, and other large farm animals [15–18] (Figure 2). The differentiated B cells in those organisms essentially rely on somatic gene conversion to generate Ig diversity, especially in the hyper-variable region [19,20]. The resulting sequence divergence ensures the generation of a wide range of functional Ig repertoires.

In the chicken pre-B cell line DT40 [21], Ig light chain (IgL) and heavy chain (IgH) contain only a single functional V-J segment in one allele. Successive rounds of templated and unidirectional transfer of short DNA stretches are introduced from the upstream pseudo-V segment clusters to the functional V region, leading to an increase in the sequence divergence of Ig V segments [21]. Therefore, the upstream pseudogene cluster functions only as a “template” for gene conversion, and the original copy remains unaltered [22]. Interestingly, in the absence of pseudogene templates, gene conversion can no longer take place. Elimination of the IgL upstream pseudogene array hampered gene conversion at the IgL V region, but enhanced somatic hypermutation [23]. Therefore, the presence of multiple and different template sequences is critical for the diversifying gene conversion.

Some pathogenic microorganisms, such as the spirochete *Borrelin hermsii* and African trypanosomes, have similar types of gene conversion-based diversification for genes encoding outer membrane proteins. The *Borrelin* variable major protein and the *Trypanosoma* variant surface glycoprotein genes are generated by gene conversion processes from donor pseudogenes [24,25]. Genetic alterations based on gene conversion allow such organisms to evade the immune system of their hosts.

## 3. DNA Modifying and Recombination Enzymes Involved in Ig Gene Conversion

The Ig gene conversion process involves functions of various DNA-related enzymes including activation-induced cytidine deaminase (AID) [26,27] and Rad51 paralogs (Rad51B, Rad51C, Rad51D, XRCC2, XRCC3) [28], as described below.

AID was identified as an RNA-editing enzyme, based on its similarity to the RNA-editing enzyme APOBEC-1 [29]. AID was first shown to be essential for class-switch recombination and somatic hypermutation (mutations specifically introduced in hypervariable regions of Ig loci) in mice and human [29,30]. Thus, AID is responsible for no less than three different types of DNA diversifying events in Ig loci. Indeed, deletion of the AID gene in B cells conferred loss of gene conversion, class-switch recombination, and somatic hypermutation.

AID is a cytidine deaminase that converts cytidines to uracils [31,32]. Such uracils are supposed to be eliminated by uracil DNA glycosylase (UNG), thereby causing DNA lesions that may initiate homologous recombination. In fact, introduction of the UNG inhibitor (UGI) coding sequence into DT40 cells led to a reduction of gene conversion frequency [33]. The same group later confirmed that DNA single- or double-strand breaks induced at AID-dependant uracils are involved in gene conversion [34]. These observations were supported by the fact that deletion of the UNG gene in DT40 markedly reduced gene conversion rates [35]. Interestingly, transgenic mice constitutively expressing AID tend to generate cancers in various tissues [36]. In addition, the infection of *Helicobacter pylori* affects genome integrity of gastric cells due to an increased expression of AID, which leads to the accumulation of mutations in the p53 tumor suppressor gene [37]. Upregulation of AID was observed in gastritis with *H. pylori* infection and gastric cancer tissues [37].

Rad51 paralogs are important for DNA homology search and the formation of heteroduplexes, which are essential intermediates for gene conversion between the donor and recipient DNA molecules [38]. Somatic hypermutation is relatively rare at Ig loci in DT40, as compared to gene conversion. However, in the absence of Rad51 paralogs, Ig gene conversion is abolished in chicken DT40 cells [28] and somatic hypermutation is, in turn, highly enhanced. As mentioned previously, a similar shift from gene conversion to hypermutation was observed at the IgL V region in DT40 where the IgL upstream pseudogene templates were eliminated [23]. In the absence of the template pseudogenes, gene conversion can no longer take place, and in turn, hypermutation becomes activated. This is because AID is involved in the initiation steps of both gene conversion and hypermutation. These two diversification pathways may differentiate in the subsequent DNA damage repair steps.

## 4. Possible Mechanisms for Donor/Recipient Choice of Gene Conversion

Targeting of AID to the Ig variable segments should be critical for establishment of the recipient choice for Ig gene conversion. Precisely how AID is targeted to Ig loci remains an open question, but we would like to speculate based on present observations.

Firstly, sequence-specific DNA binding proteins may be responsible for the specific recruitment of AID to the variable regions. Indeed, E2A and NF-kappaB family transcription factors are notably involved in Ig gene conversion [39–43]. Though transcription factors may be mainly involved in chromatin modifications, this raises the possibility that *cis*-acting elements for gene conversion and hypermutation may be present at Ig loci or in their close proximity. In fact, deleting the genomic DNA sequence including the flanking region of IgL locus in DT40 abolished both gene conversion and hypermutation of the IgL gene [44]. Step-wise deletions of the IgL locus revealed that a 9.8 kilobase flanking DNA sequence located downstream of the IgL transcription start site is necessary for the hypermutation activity [45]. This sequence, called “*cis*-acting diversification activator” (DIVAC), is sufficient for AID-mediated hypermutation, since its insertion to non-Ig loci predispose neighboring transcription units to hypermutation.

Secondly, transcriptional activity in the Ig allele is involved in the loading of AID. Each of the IgL and IgH alleles in chicken B cells has two homologous (paternal and maternal) copies. Only one of them is recombined by V(D)J recombination, which leads to the elimination of the transcriptional silencer sequence. These transcription-active alleles, unlike non-transcribed alleles, can function as the recipients of gene conversion. This suggests a link between transcription and gene conversion mediated by AID. Recent *in vitro* experiments revealed that AID is efficiently targeted to relaxed or single-stranded DNA regions in nucleosomes only during transcription [46]. Thus, transcription along the active V regions may facilitate the recruitment of AID. However, transcription *per se* is a genome-wide event and not restricted to the active V regions. Therefore, the site of AID loading may be determined by the combination of transcription, DNA accessibility, and other factors.

## 5. Chromatin and Epigenetic Control of Gene Conversion

It has been suggested that local chromatin structure and epigenetic marks play pivotal roles in the regulation of the Ig gene conversion. Histone acetylation, in particular, has been demonstrated to regulate somatic recombination of Ig and T-cell receptor loci such as V(D)J recombination [47–49] and class-switch recombination [50,51] in the immune system of vertebrates. Importantly, the Ig V regions in the recombination-active allele exhibited higher histone acetylation levels, when compared to the recombination in inactive alleles [52]. Additionally, gene conversion frequency increased when chicken DT40 cell lines were treated with trichostatin A (TSA), an inhibitor for one of the histone deacetylases (HDACs) [52]. Similar results were obtained by knocking out the native HDAC1 and HDAC2 genes in DT40 [53,54]. It should be noted that the HDAC2 knockout cells exhibit a rather broader distribution of gene conversion with relatively shorter tracts. Thus, histone modification may regulate the length and position of DNA sequence alterations.

The Ig pseudogene array in DT40 is also characterized by higher levels of histone modifications associated with active chromatin. Artificial tethering of a heterochromatin protein HP1-lactose repressor to lactose operators integrated in the pseudo-V array diminished histone acetylation within the pseudo-V region, leading to repression of gene conversion but enhancement of hypermutation [55]. These results suggest that unidirectional Ig gene conversion requires an open chromatin state in both donor pseudogenes and recipient V regions.

## 6. Control of DNA Sequence Diversity by Gene Conversion

**Figure 3:**
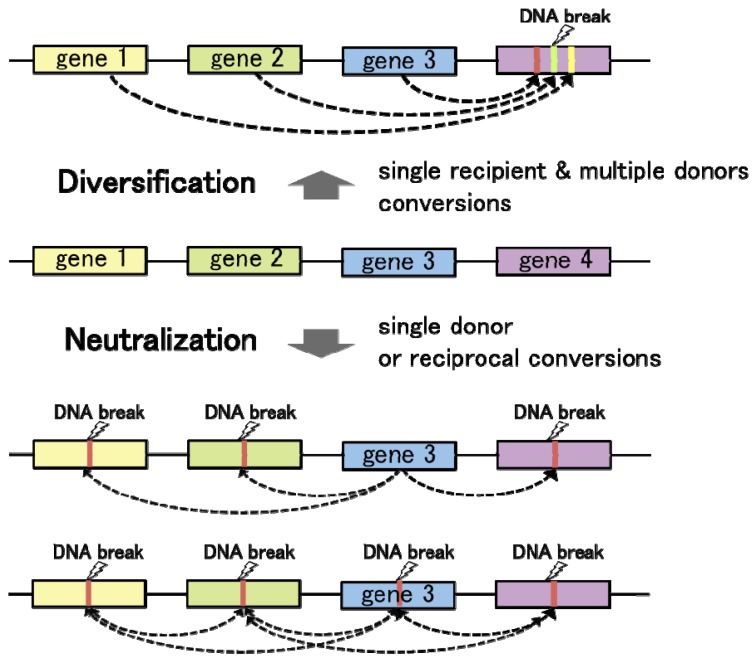
Regulation of genome alteration and integrity by gene conversion. The donor/recipient choice for gene conversion among homologous DNA sequences is determined by the localization of recombination-initiating DNA lesions, which are introduced under the influence of epigenetic marks such as histone acetylation, local chromatin structure, and transcriptional activity (shown as broken wavy lines). Gene conversion with a single template sequence neutralizes the divergence of the homologous DNA sequences. Adversely, the convergent gene conversion from multiple different DNA sequences results in diversification of the recipient locus.

Convergent gene conversion from multiple different template sequences to a single recipient locus contributes to genetic divergence at the recipient allele (Figure 3). On the other hand, gene conversion from a single template to multiple (or single) recipient loci tends to diminish sequence variation among homologous DNA sequences. Hence, regulation of the donor/recipient choice may be critical for the function of gene conversion in diversification and neutralization of homologous DNA sequences. As observed in chicken Ig loci, targeted delivery of the proteins responsible for DNA lesions locally triggers homologous recombination at the recipient loci. On the one hand, introduction of artificial DNA breaks by tethering the I-SceI endonuclease to a chromosomal LINE repetitive element resulted in DSB repair by gene conversion with various endogenous L1 elements, especially with some of the most active retrotransposable elements [56]. In these cases, gene conversion works towards the diversification of genome sequences. On the other hand, in the budding yeast mating type switching, HO-endonuclease introduces a DSB at the recipient *MAT* locus, leading to “copy and paste” type transfer of genetic information from the template alleles *HML* or *HMR*. In this case, gene conversion functions to neutralize the sequence difference between the recipient and donor loci. Thus, the number of donor sequences and selection of the recipient loci govern the functions of gene conversion in genome dynamics.

What are the major determinants for the donor/recipient choice? In eukaryotes, homologous recombination control depends on the local chromatin structure. Therefore, chromatin is supposed to be a primary determinant in the donor/recipient choice of gene conversion. Importantly, epigenetic marks on chromatin, which are known to regulate local DNA accessibility, are established during differentiation, or under the influence of extracellular signals. Therefore, it is speculated that eukaryotic cells may utilize chromatin structure as an intrinsic modulator for genome maintenance and rearrangement, in response to a given environment. Moreover, transcriptional activity is often coupled with high DNA accessibility. Differences in transcriptional activities between duplicated or repetitive genes may influence the donor/recipient choice of gene conversion: increased transcription can result in a higher likelihood for a sequence to become a recipient of gene conversion. If so, duplicated genes with high levels of expression can undergo persistent diversification or neutralization by gene conversion, whereas transcriptionally silent loci would lose their ability to diversify DNA sequences at least as recipients of gene conversion. The relationship between chromatin structure and gene conversion requires further investigation, and may provide important clues to understand the regulation of genome evolution.
